# Decreased Serum Decorin Levels Are Correlated with Aortic Stiffness as Assessed Using Carotid–Femoral Pulse Wave Velocity in Patients with Peritoneal Dialysis

**DOI:** 10.3390/life15040541

**Published:** 2025-03-26

**Authors:** Yahn-Bor Chern, Po-Yu Huang, Yu-Li Lin, Chih-Hsien Wang, Jen-Pi Tsai, Bang-Gee Hsu

**Affiliations:** 1 Division of Nephrology, Department of Internal Medicine, Yuan’s General Hospital, Kaohsiung 80249, Taiwan; 2 Institute of Medical Sciences, Tzu Chi University, Hualien 97004, Taiwan; 3 Division of Nephrology, Department of Internal Medicine, Dalin Tzu Chi Hospital, Buddhist Tzu Chi Medical Foundation, Chiayi 62247, Taiwan; 4 Division of Nephrology, Hualien Tzu Chi Hospital, Buddhist Tzu Chi Medical Foundation, Hualien 97004, Taiwan; 5 School of Medicine, Tzu Chi University, Hualien 97004, Taiwan

**Keywords:** decorin, arterial stiffness, peritoneal dialysis, carotid–femoral pulse wave velocity, biomarker

## Abstract

In patients on chronic peritoneal dialysis (PD), aortic stiffness (AS) is a common cardiovascular condition that can predict cardiovascular events and mortality. Decorin is a small leucine-rich proteoglycan that plays a vital role in extracellular matrix organization and vascular remodeling. The relationship between decorin and AS in patients with PD remains unclear. We enrolled 140 patients on PD and collected their demographic, anthropometric, and biochemical data. Serum decorin levels were measured using enzyme-linked immunosorbent assay. Based on carotid–femoral pulse wave velocity (cfPWV), a diagnosis of AS was established in 42 patients (30%), who were found to be of advanced age and showed higher prevalence rates of systolic blood pressure, diabetes, hypertension, triglyceride, fasting glucose, and lower decorin levels, compared with those who had no AS. After proper adjustment for confounding factors in the multivariable logistic regression model, AS development was associated with decorin, age, and triglyceride levels. Multivariable linear regression analysis showed that decorin, when subjected to logarithmic transformation, can be viewed as a significant independent predictor of cfPWV (β = −0.289; *p* < 0.001). Low decorin level was significantly and independently associated with AS in patients undergoing chronic PD.

## 1. Introduction

Chronic peritoneal dialysis (PD) is a prevalent therapeutic modality for individuals diagnosed with end-stage renal disease (ESRD), facilitating the incremental elimination of metabolic byproducts and excess fluid via the peritoneal membrane. Despite its benefits, chronic PD increases the risk of cardiovascular (CV) mortality, which remains the leading cause of death among patients [1,2]. Among the various CV mortality predictors in the community settings and patients suffering from chronic kidney disease (CKD), aortic stiffness (AS) has been extensively studied in recent decades and has been proven to be an independent predictor of future CV diseases and all-cause mortality [3,4,5,6]. Multiple traditional and nontraditional risk factors for atherosclerotic CV diseases can contribute to AS in patients undergoing chronic PD; these include chronic inflammation, vascular calcification, volume overload, high oxidative stress, and premature arterial stiffening [7].

AS is a clinical condition characterized by augmentation of arterial wall properties, resulting in altered arterial flow and diameter and future CV diseases [8]. Since the publication of the European Society of Hypertension/European Society of Cardiology guidelines on managing arterial hypertension [9], measuring aortic pulse wave velocity (PWV) has become the gold standard for detecting AS. Among the different measurement devices and methods, the carotid–femoral PWV (cfPWV) technique is used to detect aortic AS [8]. A systematic review and meta-analysis study has validated the accuracy of utilizing cfPWV for predicting CV and all-cause mortality; in four studies that focused on patients with ESRD, cfPWV was used to diagnose AS [10].

Decorin is a small leucine-rich proteoglycan that plays a significant role in various physiological and pathophysiological processes [11], such as collagen formation, signaling networks, and interactions with extracellular matrix components, growth factors, and receptor tyrosine kinases (RTKs) [12,13]. The functions of decorin extend to immune regulation, vascular health, cancer suppression, and ocular health, among others [11,14,15,16]. Because of its anti-inflammatory, anti-oxidative, and anti-fibrotic effects, decorin can be linked to vascular health and diseases, such as AS and atherosclerosis, which are common in patients with ESRD [12,16,17]. Furthermore, the reported association between low decorin levels and more extensive peritoneal fibrosis in patients undergoing PD further strengthens the role of decorin as an anti-fibrotic agent and a potential therapeutic target in this population [18]. Table 1 summarizes currently known functions and roles of decorin. However, previous studies have not investigated the association between serum decorin level and AS in patients with chronic PD, which is the focus of this paper.

The objective of the present study was to examine the correlation between serum levels of decorin and AS, as measured by cfPWV, in chronic PD patients. Identifying novel biomarkers of subclinical vasculopathy and AS could help clinicians improve risk stratification in this vulnerable patient population. More importantly, our findings can act as a guide for future therapeutic interventions targeting vascular health in patients undergoing chronic PD. Considering decorin’s diverse involvement in vascular biology and fibrosis, clarifying its relationship with AS in this population could provide a deep understanding of possible pathophysiological mechanisms and therapeutic opportunities.

## 2. Materials and Methods

### 2.1. Patient Selection and Data Collection

A cross-sectional study was conducted at two hospitals affiliated with the Tzu Chi Medical Foundation in Taiwan. The research protocol received approval from the Research Ethics Committee of Hualien Tzu Chi Hospital, Buddhist Tzu Chi Medical Foundation, with the approval number of IRB108-219-A. We enrolled 140 patients with ESRD undergoing maintenance PD for ≥6 months between January 2020 and October 2021. Enrollment was restricted to patients under the care of participating nephrologists. Written informed consent was obtained from each patient prior to enrollment. Patients were excluded if they had the following conditions: active infection, recent acute coronary syndrome, heart failure with acute decompensation, stroke, active malignancy, or history of limb amputation, as well as those unwilling to consent.

We reviewed each patient’s medical records to collect demographic and biochemical data, such as age, sex, ESRD etiology, PD vintage and modalities, and the prescribed long-term anti-hypertensive and lipid-lowering medications. Small solute clearance indicators, including overall and weekly peritoneal creatinine clearance and weekly fractional clearance index for urea (Kt/V), were also obtained from the charts. The most recently available data on PD adequacy before enrollment were selected. Residual renal function was determined by both the 24 h urinary creatinine clearance and Kt/V, which were calculated according to the recommendations of the Taiwan Society of Nephrology.

For blood pressure assessment, patients were asked to sit quietly for 10 min before a trained staff member started measurements using a sphygmomanometer with calibrated mercury and a cuff of appropriate size. A diagnosis of hypertension was made if a patient’s systolic blood pressure (SBP) was ≥140 mm Hg or diastolic blood pressure (DBP) was ≥90 mm Hg, or in patients who had taken anti-hypertensive medications within the most recent 2-week period. Diabetes mellitus (DM) was diagnosed if the patient had a fasting plasma glucose level of ≥126 mg/dL or was already on long-term anti-diabetic therapy.

### 2.2. Anthropometric and Biochemical Assessments

Body mass index (BMI) was determined by the quotient of body weight (in kilograms) and the square of height (in meters squared). We collected 5 mL of fasting blood sample from each patient when dialysate fluid was completely drained and before new dialysate fluid was instilled in the morning; 0.5 mL of this sample was for measuring complete blood count using Sysmex SP-1000i (Sysmex American, Mundelein, IL, USA), and the residual 4.5 mL was promptly centrifuged at 3000× *g* for 10 min. The collected serum was processed in an autoanalyzer (Siemens Advia 1800; Siemens Healthcare, Henkestr, Germany) to measure the levels of total cholesterol, triglyceride, fasting glucose, albumin, blood urea nitrogen, creatinine, total calcium, and phosphorus. The decorin and intact parathyroid hormone (iPTH) serum concentrations were obtained by using an enzyme-linked immunosorbent assay kit (Abcam, Waltham, MA, USA and IBL International GmbH, Hamburg, Germany, respectively).

### 2.3. Assessment of Carotid–Femoral Pulse Wave Velocity as an Indicator of Arterial Stiffness

The PWV of each patient was measured using the SphygmoCor XCEL device (AtCor Medical, Sydney, NSW, Australia), which has a system that can verify the tonometer, femoral cuff, and connecting tubing to improve measurement accuracy. We followed the device manual for troubleshooting, calibration, and quality control. Before each measurement, the participants were asked to rest and relax in a supine position for approximately 10 min and to avoid talking or moving their limbs during the process. After measuring and recording brachial SBP and DBP, the inflatable cuff was subsequently reinflated for five seconds to have arterial waveforms captured. Then, trained staff members placed a tonometer on the carotid pulsation point, which was identified by palpation. When a regular carotid waveform was detected, the SphygmoCor XCEL device would automatically inflate the femoral cuff encircling a participant’s thigh to record the corresponding waveforms of a femoral artery at the same time. The distance traveled by the pulse was estimated by deducting the distance separating the carotid pulse from the suprasternal notch from the distance between the pulsation of a patient’s femoral artery and the suprasternal notch. The calculation of cfPWV was then achieved by dividing the distance of pulse transmission by the pulse’s traveling time in meters per second between the two anatomical sites. To reduce bias, each patient’s cfPWV was assessed twice, and the average number was recorded for further analysis. Based on current clinical guidelines [19], individuals with a cfPWV value of ≥10 m/s were diagnosed with AS.

### 2.4. Statistical Analysis

Power analyses were conducted to determine whether the enrolled patient number was sufficient to establish a significant correlation. First and foremost, given a detection of a correlation coefficient of approximately 0.3 between serum decorin levels and cfPWV, with an alpha value of 0.05 and a power of 80%, a total of at least 85 patients should be included in the study. If the power is raised to 90%, then at least 113 patients should be included to determine that the correlation coefficient is different from zero.

All data were examined for normality before linear regression was applied. The Kolmogorov–Smirnov test was employed to assess the normal distribution characteristics of continuous variables. For variables that were normally distributed, we reported them as mean ± SD and made a comparison between the AS and non-AS groups using an unpaired Student’s *t*-test, whereas for those that did not meet normality, we compared them by applying the Mann–Whitney U test. Categorical variables were displayed as numerical representations and percentages and were compared between groups using the chi-square test. Variables that showed significant differences between groups were identified as adjustable factors and were then included in a multivariable logistic regression model to identify independent predictors of AS. To address potential multicollinearity among independent variables, we assessed the variance inflation factor (VIF). Variables with VIF values greater than 10 were considered highly collinear, while VIF values below 5 indicated no significant collinearity issues.

Univariable analyses identified age, SBP, fasting glucose, triglyceride, and decorin levels, and the presence of DM and hypertension as adjustable factors. Simple and multivariable regression analyses were conducted to ascertain the variables that were correlated independently with cfPWV; for those variables with skewed distributions (i.e., PD duration, triglyceride, fasting glycemic level, iPTH, and decorin), logarithmic transformation (log) was applied beforehand. Results were reported in the form of odds ratio (OR) accompanied by a 95% confidence interval (CI). The Windows-version software IBM SPSS Statistics 19.0 (IBM, Armonk, NY, USA) was applied for all statistical analyses. A receiver operating characteristic (ROC) curve analysis (MedCalc Software Ltd., version 22.019, Ostend, Belgium) was performed to determine the optimal serum decorin cutoff value for differentiating between the AS and non-AS groups and to generate boxplots and scatter plots.

## 3. Results

The baseline characteristics of the 140 patients receiving chronic PD therapy who were included in this study are presented in Table 2. The treatment modality was continuous ambulatory PD in 40.0% (56 patients) and automated PD in 60% (84 patients). The prevalence rates of DM and hypertension were 40.7% and 75.0%, respectively. The median PD vintage was 50.82 months (interquartile range, 25.00–87.12 months). Based on cfPWV of ≥10 m/s, AS was diagnosed in 42 patients. In comparison to the control cohort, the AS group was significantly older (62.36 ± 9.74 years vs. 57.28 ± 14.30 years, *p* = 0.037); had significantly higher SBP (*p* = 0.023) and higher concentrations of fasting glucose (*p* = 0.017) and triglyceride (*p* = 0.003); significantly lower serum decorin level (*p* < 0.001); and significantly higher prevalence of DM (59.5% vs. 32.7%, *p* = 0.003) and hypertension (88.1% vs. 69.4%, *p* = 0.019). On the other hand, no significant differences were identified between the AS and non-AS groups in the following clinical factors: sex distribution, BMI, and DBP; as well as hemoglobin, total serum cholesterol, albumin levels, blood urea nitrogen, creatinine concentrations, total calcium, levels of phosphorus, and iPTH; weekly Kt/V, peritoneal Kt/V, total weekly creatinine clearance, and peritoneal creatinine clearance; and frequency of intake of anti-hypertensive medications, such as angiotensin receptor blockers, beta-blockers, and calcium channel blockers, or statins.

Multivariable logistic regression analysis (Table 3) revealed that low serum decorin level was significantly associated with decreased risk for AS (OR 0.863, 95% CI 0.784–0.949, *p* = 0.002). Moreover, AS risk had a significant positive correlation with increasing age (OR 1.053, 95% CI 1.013–1.095, *p* = 0.009) and a modest but significant association with high triglyceride level (OR 1.008, 95% CI 1.002–1.014, *p* = 0.009).

On simple regression analysis (Table 4), the clinical factors that significantly correlated with cfPWV among patients with chronic PD were age (*r* = 0.248, *p* = 0.001); SBP (*r* = 0.254, *p* = 0.002); log-triglyceride (*r* = 0.233, *p* = 0.006); log-glucose (*r* = 0.192, *p* = 0.023); and log-decorin (*r* = –0.264, *p* = 0.002). In the context of multivariate stepwise linear regression analysis, we identified independent predictors in terms of an increased cfPWV as advanced age (β = 0.330, adjusted R^2^ = 0.074, *p* < 0.001); high SBP (β = 0.259, adjusted R^2^ = 0.065, *p* = 0.001); elevated log-triglyceride (β = 0.215, adjusted R^2^ = 0.042, *p* = 0.004); and low log-decorin (β = –0.289, adjusted R^2^ = 0.084, *p* < 0.001). Among the analyzed variables, log-decorin was significantly and negatively correlated with cfPWV. The results indicated that a low decorin level was associated with a relatively high risk for AS. Scatter plots to further evaluate the relationships between cfPWV and these variables (i.e., age, SBP, log-triglyceride, and log-decorin) are shown in Supplementary Figure S1.

**Figure 1:**
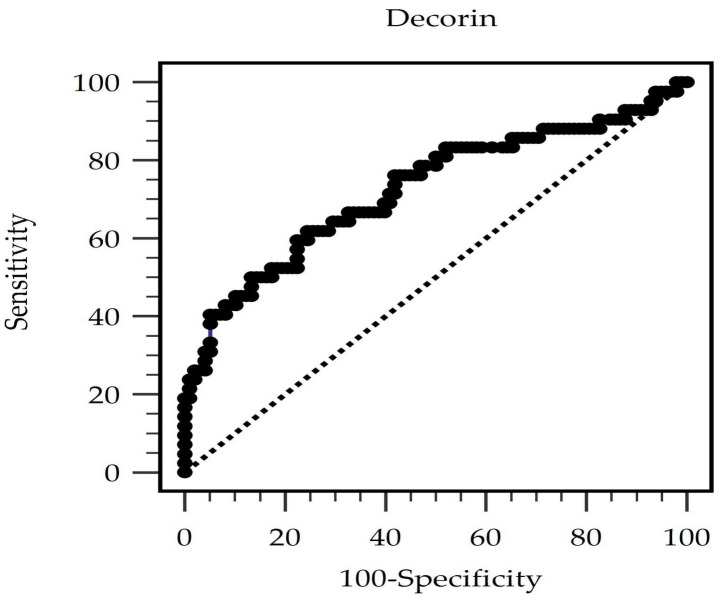
The area under the receiver operating characteristic curve delineates the diagnostic power of decorin levels for predicting arterial stiffness among peritoneal dialysis patients.

An ROC curve was plotted to calculate the area under the curve (AUC) to evaluate the diagnostic accuracy of serum decorin level in predicting AS among patients on PD. ROC curve analysis (Figure 1) showed that the optimal decorin threshold of 16.91 ng/mL had an AUC of 0.725 (95% CI 0.625–0.826, *p* < 0.0001) in predicting AS among patients on PD, with 61.90% sensitivity, 75.51% specificity, 52.00% positive predictive value, and 82.22% negative predictive value.

## 4. Discussion

This study investigated the relationship between serum decorin level and AS among patients with chronic PD. The key results were as follows: (1) independent associations of clinical factors, such as advanced age, high SBP and triglyceride levels, and decreased decorin level, with increased AS, and (2) the negative relationship between log-decorin and cfPWV.

In previous reports, the clinical factors identified by multivariate analyses to be correlated with AS were advanced age [20,21]; genetic predisposition [22]; family history of diabetes and/or hypertension [23,24]; pre-existing atherosclerotic CV diseases [25,26]; and traditional CV risk factors, such as DM [27,28], hypertension [20,21], dyslipidemia [29,30], glucose intolerance [27], obesity [31], sedentary lifestyle [32], and smoking [33]. In agreement with our results, aging and elevated blood pressure were previously shown to play a significant role in evaluating the degree of AS [7,34,35]. In aging-associated AS, endothelial cell senescence and aging-related vascular dysfunction, such as impaired myogenic and autoregulatory responses, may partly explain the relatively high prevalence of various vasculopathies in the elderly [36]. In addition, the propensity of older individuals to have more comorbidities increases their susceptibility to developing AS. As another dominant contributor to AS, hypertension mainly exerts its effects through vascular remodeling secondary to shearing forces on vascular endothelial and smooth muscle cells [34]. Meanwhile, previous studies have shown that hypertriglyceridemia, in conjunction with other factors, increases the probability of AS. In fact, a meta-analysis confirmed that individuals with high triglyceride–glucose index, which is a marker of insulin resistance, had a relatively high risk of developing AS [37]. Therefore, our results on the independent associations of older age and high SBP with elevated cfPWV agreed with the current evidence.

Decorin belongs to the family of small leucine-rich repeat proteoglycans and has anti-fibrotic, anti-oxidative, anti-inflammatory, tissue repair, and context-dependent anti-angiogenic properties [16,38]. The current study focused on the association of decorin with endothelial function and vascular health, which are putative pathways to the development of AS. The potential relationships between decorin and AS may be accounted for by the aforementioned properties and underlying protective ability of decorin against AS anti-inflammatory [39], anti-angiogenic [40,41], anti-oxidative [40], and anti-fibrotic actions [42] and regulation of vascular remodeling. In a review article, Vu et al. illustrated the main biological action of decorin by demonstrating that the currently studied interactome was responsible for extracellular matrix organization and response to growth factors [16]. The functions of decorin could be partly accomplished by binding and downregulating RTKs, subsequently leading to inflammation and fibrosis secondary to a variety of downstream changes in the levels of cellular transmitters or receptors, such as transforming growth factor β and epidermal growth factor receptor; cytokines, such as tumor necrosis factor α; and hormones, such as insulin-like growth factor [16,41]. Furthermore, in patients with chronic PD, low decorin level was found to be associated with decreased intracellular adhesion molecules of monocytes and the resulting decrease in monocyte activation and inflammation [18]. Therefore, the putative explanation for the association between low decorin level and AS in this study could be the reduced anti-inflammatory, anti-oxidative, and anti-fibrotic protective effects of decorin.

In addition to AS, atherosclerosis can be linked to decorin in patients undergoing chronic PD. Low decorin level was found to be associated with vascular smooth muscle calcification, inflammation, lipid accumulation, intimal hyperplasia, angiogenesis, and extracellular matrix remodeling through microarray analysis [12,38,43]. AS and atherosclerosis are strong predictors of CV events and all-cause mortality in this vulnerable population. Considering the multifaceted roles of decorin in various signaling pathways and its potential as a therapeutic target in vascular diseases, this study highlighted the need to further elucidate the mechanistic relationships among decorin, AS, and atherosclerosis in patients with CKD/ESRD on dialysis therapy.

AS in CKD patients is influenced by a variety of renal- or inflammatory-specific biomarkers related to both CKD–mineral and bone disease (CKD–MBD) and non-CKD–MBD pathways. Among the biomarkers related to CKD–MBD, fibroblast growth factor-23 (FGF-23), osteoprotegerin (OPG), calcium, phosphate, and parathyroid hormone (PTH) have been extensively studied. Elevated FGF-23 levels are strongly linked to arterial calcification, increased pulse wave velocity, and cardiovascular mortality in CKD patients [44,45]; however, conflicting results were presented by some studies, which might be caused by confounding factors [46]. Similarly, a key regulator of bone metabolism, OPG, was found to be associated with vascular calcification and independently predicts cardiovascular outcomes [47,48]. Other biomarkers associated with CKD–MBD, including calcium, phosphate, and PTH, have been linked to vascular calcification and cardiovascular mortality [49,50,51]. Some non-CKD–MBD biomarkers, such as asymmetric dimethylarginine (ADMA), resistin, and C-reactive protein (CRP), were demonstrated to be related to a number of cardiovascular diseases. Of these, ADMA acts as a nitric oxide (NO) synthase inhibitor that could cause endothelial dysfunction and atherosclerosis by reducing NO concentrations [52]. Moreover, studies have shown that ADMA correlates with arterial stiffness and atherosclerosis in CKD populations, which can be observed even in the condition’s early stages [52,53]. Our previous study found that an inflammatory adipokine, resistin, is associated with aortic stiffness in non-dialytic CKD patients [54]. Finally, CRP has been established as a systemic inflammation marker that could be implicated in arterial stiffening and predicts cardiovascular mortality [55]. Despite the above findings, it is essential to address the fact that using a single biomarker to predict the presence of AS is of limited value due to the influences of confounding factors, such as concomitant infection, malignancy, impacts of dietary and lifestyle issues, and medication effects, and concerns about cost-effectiveness. Furthermore, a causal link between these biomarkers and AS is difficult to establish because of CKD patients’ complex and bidirectional pathogenic processes regarding AS development and progression. Our study centers on decorin, which has been found to have anti-fibrotic and vascular-protective properties. However, compared to other biomarkers, its role in predicting arterial stiffness in patients with CKD is not fully explored and is expected to be less affected by treatments or interventions targeting the CKD–MBD pathway. Thus, an investigation into the association of serum decorin levels with arterial stiffness in patients with CKD may provide new insights into the pathogenic processes leading to vascular and cardiac complications in these patients.

In terms of clinical implications, this study provides a novel biomarker for potential integration into the current risk stratification system regarding cardiovascular diseases and could facilitate further therapeutic strategies in patients undergoing chronic PD, which might include guiding doctor–patient discussions on cardiovascular risks, enhancing lifestyle interventions in high-risk patients, and guiding decision-making for pharmacologic intervention or further invasive testing of other atherosclerotic cardiovascular diseases. Additionally, the combination of measuring decorin levels and performing standard diagnostic tools such as pulse wave velocity measurements may be beneficial to the early detection of subclinical vasculopathy, which can then allow physicians to implement personalized and patient-centered treatment strategies in a timely manner. Several interventions could then be provided to mitigate the patient’s overall cardiovascular burden, such as optimizing prescriptions of dialysis, managing CKD–MBD aggressively, and aiming at intensive targets for underlying conditions like diabetes, hypertension, and dyslipidemia in patients at higher cardiovascular risk. To conclude, the idea of measuring decorin in combination with traditional cardiovascular risk assessments provides healthcare professionals a chance to better understand the multifactorial mechanisms behind arterial stiffness and improve long-term outcomes in chronic PD patients.

This study had several limitations. First, the relatively small sample size and recruitment of patients from a limited number of centers restricted us from generalizing our findings to all patients with chronic PD. Second, because of the cross-sectional study design, establishing direct causality between decorin level and AS is challenging. Third, serum decorin level was measured only once per participant; therefore, variations over time or under different conditions may not have been captured. Furthermore, although inflammation may have contributed to AS progression, this study did not measure biomarkers related to inflammation, such as C-reactive protein and interleukins. Future longitudinal research with larger cohorts could provide further insights into the relationship between these inflammatory markers and AS in patients with long-term PD. Finally, although decorin demonstrated an acceptable AUC of 0.725, the complexity and multifactorial nature of the pathogenic mechanisms of AS in patients with chronic PD require future studies on additional biomarkers with more substantial predictive power. This can improve the clinical utility of AS prediction in this patient population.

## 5. Conclusions

This study demonstrated that low serum decorin levels independently predicted the presence of AS in patients with chronic PD, as measured by cfPWV. Our results suggest that decorin could serve as an applicable biomarker for stratifying cardiovascular risk and may be an add-on marker that can facilitate existing diagnostic tools in the early detection of asymptomatic vascular diseases in this vulnerable population. To conclude, by assessing decorin levels together with traditional cardiovascular risk factors, clinicians may better understand the complexity of interactions between arterial stiffness and various cardiovascular risk factors, which can, in turn, improve long-term cardiovascular outcomes of patients undergoing chronic PD.

The limitations of this study should be mentioned, which include a relatively small sample size with patients recruited from two centers in the same country, the inability to confirm the causal connection between levels of decorin and AS because of the cross-sectional design, a single-time-point measurement of decorin, and not including other inflammatory markers. Although we have identified decorin’s predictive ability in this study, additional biomarkers with more remarkable predictive power should be explored regarding the complexity of pathogenic mechanisms contributing to AS. Further research involving longitudinal studies with larger sample sizes is warranted to examine whether decorin could serve as a predictive tool and therapeutic target for improving vascular health in the chronic PD population.

## Figures and Tables

**Table 1:** Key functions and roles of decorin summarized based on current published studies.

Proposed Role	Description	References
Interacting With Extracellular Matrix	Involved in collagen formation and interactions with extracellular matrix components	[11,12]
Signaling Networks	Participates in signaling networks and interacts with growth factors and receptor tyrosine kinases	[12,13]
Immune Regulation	Plays a role in regulating immune responses	[11,14]
Vascular Health	Contributes to vascular health through anti-inflammatory, anti-oxidative, and anti-fibrotic effects	[11,16]
Suppression of Cancer	Exhibits cancer suppression properties by inhibiting metastasis and angiogenesis	[11,15]
Ocular Health	Involved in eye diseases caused by ischemic or fibrotic mechanisms	[11,14]
Linked to Vascular Diseases	Related to cardiovascular diseases by involvement in inflammation and changes in endothelial phenotypes	[12,16,17]
Decrease in Peritoneal Fibrosis	Associated with reduced peritoneal fibrosis in peritoneal dialysis patients	[18]

**Table 2:** The characteristics of patients undergoing chronic peritoneal dialysis with or without the presence of arterial stiffness.

Clinical Variables	All Enrolled Patients (*n* = 140)	Control Group (*n* = 98)	Arterial Stiffness Group (*n* = 42)	*p*-Value
Age (years)	58.80 ± 13.27	57.28 ± 14.30	62.36 ± 9.74	0.037 *
Peritoneal dialysis vintage (months)	50.82 (25.00–87.12)	48.96 (23.76–84.87)	51.72 (26.23–103.00)	0.481
Body mass index (kg/m^2^)	25.10 ± 4.15	24.85 ± 4.33	25.67 ± 3.68	0.287
Carotid–femoral PWV (m/s)	9.34 ± 1.66	8.51 ± 1.01	11.28 ± 1.17	<0.001 *
Systolic blood pressure (mmHg)	149.24 ± 21.94	146.50 ± 21.15	155.64 ± 20.26	0.023 *
Diastolic blood pressure (mmHg)	84.59 ± 14.81	83.41 ± 15.60	87.36 ± 12.52	0.149
Hemoglobin (g/dL)	9.54 ± 1.44	9.61 ± 1.29	9.39 ± 1.74	0.407
Total cholesterol (mg/dL)	164.36 ± 41.53	163.90 ± 44.01	165.45 ± 35.54	0.840
Triglyceride (mg/dL)	128.50 (82.25–183.75)	122.00 (74.00–171.75)	138.50 (110.25–225.25)	0.003 *
Fasting glucose (mg/dL)	103.00 (92.25–123.00)	101.00 (91.00–116.50)	111.00 (94.75–155.75)	0.017 *
Albumin (mg/dL)	3.56 ± 0.35	3.56 ± 0.32	3.56 ± 0.41	0.941
Blood urea nitrogen (mg/dL)	65.17 ± 22.04	66.07 ± 22.50	63.07 ± 21.02	0.462
Creatinine (mg/dL)	10.97 ± 3.03	10.94 ± 3.17	11.04 ± 2.72	0.863
Total calcium (mg/dL)	9.43 ± 0.78	9.38 ± 0.74	9.53 ± 0.87	0.312
Phosphorus (mg/dL)	5.09 ± 0.85	5.12 ± 0.85	5.03 ± 0.84	0.562
Intact parathyroid hormone (pg/mL)	237.80 (93.35–482.03)	237.80 (104.04–462.80)	232.21 (80.43–526.48)	0.757
Decorin (ng/mL)	18.57 (16.03–23.30)	20.34 (1694–23.74)	16.10 (13.98–19.55)	<0.001 *
Weekly Kt/V	2.05 ± 0.41	2.05 ± 0.43	2.03 ± 0.38	0.826
Peritoneal Kt/V	1.86 ± 0.46	1.85 ± 0.49	1.88 ± 0.39	0.774
Total clearance of creatinine (L/week)	57.78 ± 16.77	56.85 ± 17.01	59.96 ± 16.19	0.316
Peritoneal clearance of creatinine (L/week)	47.65 ± 13.26	46.73 ± 14.30	49.79 ± 10.27	0.211
Female, *n* (%)	71 (50.7)	53 (54.1)	18 (42.9)	0.223
Diabetes, *n* (%)	57 (40.7)	32 (32.7)	25 (29.5)	0.003 *
Hypertension, *n* (%)	105 (75.0)	68 (69.4)	37 (88.1)	0.019 *
CAPD, *n* (%)	56 (40.0)	42 (42.9)	14 (33.3)	0.292
ARB use, *n* (%)	90 (64.3)	60 (61.2)	30 (71.4)	0.248
β-blocker use, *n* (%)	47 (52.9)	53 (54.1)	21 (50.0)	0.658
CCB use, *n* (%)	86 (61.4)	61 (62.2)	25 (59.5)	0.762
Statin use, *n* (%)	45 (32.1)	30 (30.6)	15 (35.7)	0.554

Values of continuous variables are presented as mean ± standard deviation subsequent to evaluation via Student’s *t*-test; non-normally distributed variables are expressed as median and interquartile range following assessment by the Mann–Whitney U test; categorical values are conveyed as number (%) and analyzed via the chi-square test. (Abbreviations: PWV, pulse wave velocity; CAPD, continuous ambulatory peritoneal dialysis; Weekly Kt/V, weekly fractional clearance index for urea; ARB, angiotensin receptor blocker; CCB, calcium channel blocker.) * *p* < 0.05 was considered statistically significant.

**Table 3:** Multivariable logistic regression analysis of the clinical variables correlated to arterial stiffness in patients undergoing peritoneal dialysis.

Clinical Variables	Odds Ratio	95% Confidence interval	*p*-Value
Decorin, 1 ng/mL	0.863	0.784–0.949	0.002 *
Age, 1 year	1.053	1.013–1.095	0.009 *
Triglyceride (mg/dL)	1.008	1.002–1.014	0.009 *
Systolic blood pressure, 1 mmHg	1.010	0.987–1.033	0.417
Glucose, 1 mg/dL	1.003	0.991–1.016	0.592
Diabetes, present	1.763	0.639–4.864	0.274
Hypertension, present	3.453	0.949–12.559	0.060

The analysis of the data was conducted by applying the multivariate logistic regression analysis, incorporating variables such as the presence of diabetes, hypertension, age, systolic blood pressure measurements, fasting glycemic values, triglyceride levels, and decorin concentrations. * *p* < 0.05 was considered statistically significant.

**Table 4:** Correlation between clinical variables and values of carotid–femoral pulse wave velocity levels in chronic peritoneal dialysis patients.

Clinical Variables	Carotid–Femoral Pulse Wave Velocity (m/s)				
	Univariable Regression		Multivariable Regression		
	*r*	*p*-Value	β	Adjusted R^2^ Change	*p*-Value
Age (years)	0.248	0.001 *	0.330	0.074	<0.001 *
Body mass index (kg/m^2^)	0.154	0.069	–	–	–
Log-PD duration (months)	0.040	0.639	–	–	–
SBP (mmHg)	0.254	0.002 *	0.259	0.065	0.001 *
DBP (mmHg)	0.144	0.090	–	–	–
Hb (g/dL)	0.013	0.877	–	–	–
Total cholesterol (mg/dl)	−0.050	0.559	–	–	–
Log-triglyceride (mg/dL)	0.233	0.006 *	0.215	0.042	0.004 *
Log-glucose (mg/dL)	0.192	0.023 *	–	–	–
BUN (mg/dL)	−0.119	0.161	–	–	–
Cr (mg/dL)	−0.062	0.467	–	–	–
Albumin (mg/dL)	−0.075	0.379	–	–	–
Log-iPTH (pg/mL)	−0.073	0.389	–	–	–
Total calcium (mg/dL)	0.078	0.362	–	–	–
Phosphorus (mg/dL)	−0.041	0.632	–	–	–
Log-decorin (ng/mL)	−0.264	0.002 *	−0.289	0.084	<0.001 *
Total creatinine clearance (L/week)	0.074	0.384	–	–	–
Peritoneal creatinine clearance (L/week)	0.117	0.167	–	–	–
Weekly Kt/V	−0.040	0.636	–	–	–
Peritoneal Kt/V	0.020	0.811	–	–	–

The data concerning the duration of PD, glycemic levels, triglyceride concentrations, iPTH levels, and levels of decorin revealed a skewed distribution; consequently, a logarithmic transformation was performed prior to the analysis. The data were analyzed by utilizing simple regression analyses or multivariable stepwise linear regression analysis, with the following covariates being incorporated: age, SBP, log-triglycerides, log-glucose, and log-decorin. (Abbreviations: PD, peritoneal dialysis; SBP, systolic blood pressure; DBP, diastolic blood pressure; Hb, hemoglobin; BUN, blood urea nitrogen; Cr, creatinine; iPTH, intact parathyroid hormone; Weekly Kt/V, weekly fractional clearance index for urea.) * *p* < 0.05 was considered statistically significant.

## Data Availability

The data presented in this study are available on request from the corresponding author.

## Supplementary Materials

The following supporting information can be downloaded at: https://www.mdpi.com/article/10.3390/life15040541/s1, Figure S1: The scatter plots with regression lines between the variables (A) age, (B) systolic blood pressure, (C) log-transformed triglyceride (log-triglyceride), and (D) log-decorin and the carotid–femoral pulse wave velocity.
