# Supplementary material for: Decreased Serum Decorin Levels Are Correlated with Aortic Stiffness as Assessed Using Carotid–Femoral Pulse Wave Velocity in Patients with Peritoneal Dialysis
Source: Life (Basel). 2025 Mar 26;15(4):541. doi: 10.3390/life15040541 (PMC12028904; doi:10.3390/life15040541)
Supplement: Supplementary file 1 [file life-15-00541-s001.zip › life-3513699-supplementary.pdf]

Supplementary Figure S1. The scatter plots with regression lines between the variables (A) age, (B) systolic blood pressure, (C) log-transformed triglyceride (log-triglyceride), and (D) log-decorin and the carotid-femoral pulse wave velocity.

(A)

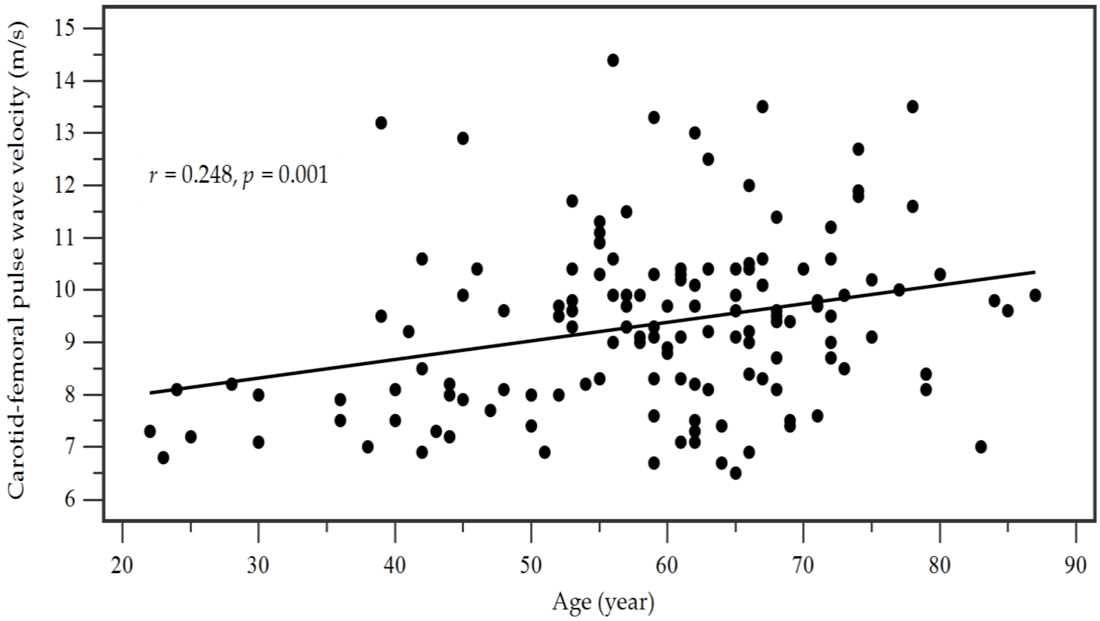

(B)

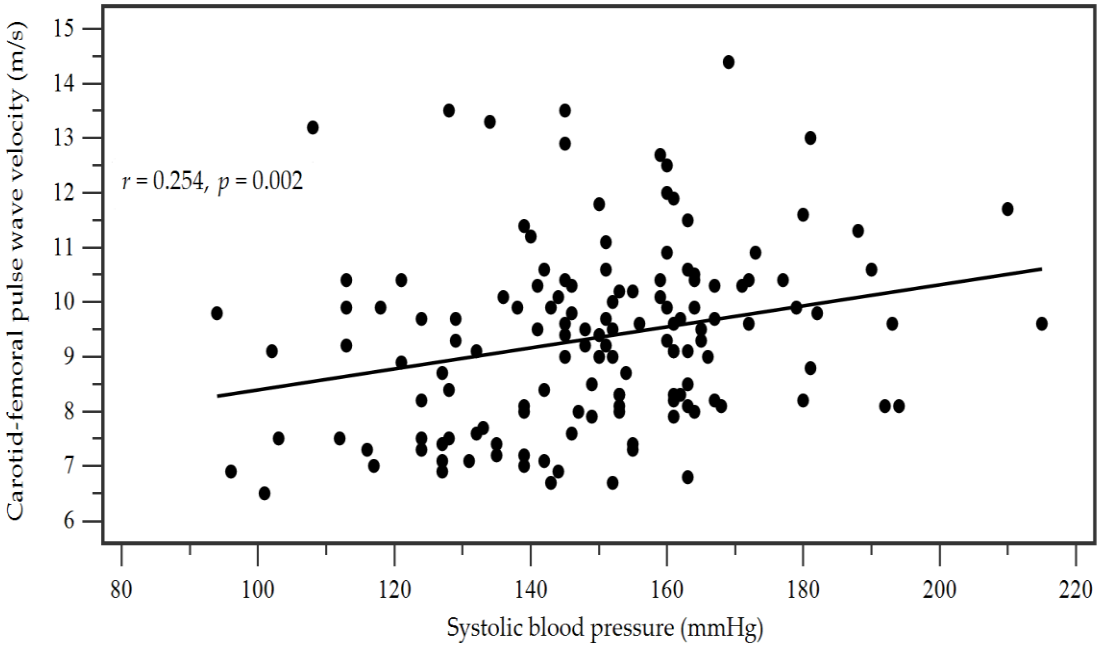

(C)

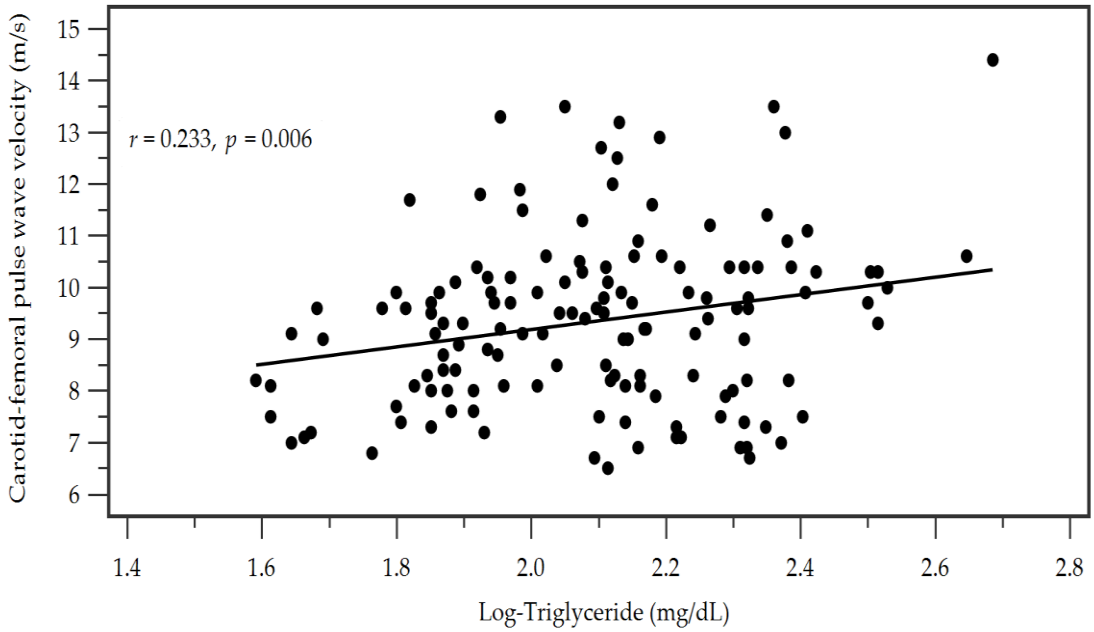
r = 0.233, p = 0.006
Carotid-femoral pulse wave velocity (m/s)
15
14
13
12
11
10
9
8
7
6
1.4
1.6
1.8
2.0
2.2
2.4
2.6
2.8
Log-Triglyceride (mg/dL)

(D)

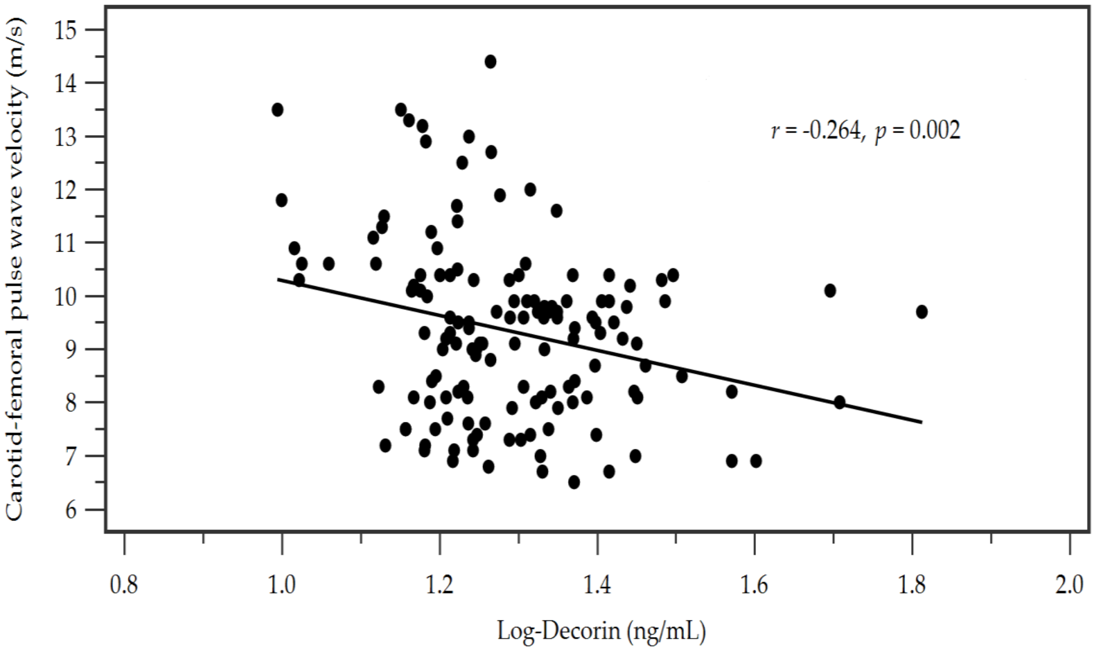
r = -0.264, p = 0.002
Carotid-femoral pulse wave velocity (m/s)
15
14
13
12
11
10
9
8
7
6
0.8
1.0
1.2
1.4
1.6
1.8
2.0
Log-Decorin (ng/mL)
